# Mechanisms by which the intestinal microbiota affects gastrointestinal tumours and therapeutic effects

**DOI:** 10.1186/s43556-023-00157-9

**Published:** 2023-11-30

**Authors:** Jikai He, Haijun Li, Jiaqi Jia, Yang Liu, Ning Zhang, Rumeng Wang, Wenhao Qu, Yanqi Liu, Lizhou Jia

**Affiliations:** 1 Central Laboratory, Bayannur Hospital, Bayannur, 015000 Inner Mongolia China; 2 Department of Gastrointestinal Surgery, Inner Mongolia Autonomous Region People’s Hospital, Hohhot, 010017 Inner Mongolia China; 3 Graduate School of Youjiang Medical University for Nationalities, No. 98 Chengcheng Road, Youjiang District, Baise City, 533000 China; 4 Department of Gastroenterology, Affiliated Hospital of Inner Mongolia Medical University, Hohhot City, 010050 Inner Mongolia China

## Abstract

The intestinal microbiota is considered to be a forgotten organ in human health and disease. It maintains intestinal homeostasis through various complex mechanisms. A significant body of research has demonstrated notable differences in the gut microbiota of patients with gastrointestinal tumours compared to healthy individuals. Furthermore, the dysregulation of gut microbiota, metabolites produced by gut bacteria, and related signal pathways can partially explain the mechanisms underlying the occurrence and development of gastrointestinal tumours. Therefore, this article summarizes the latest research progress on the gut microbiota and gastrointestinal tumours. Firstly, we provide an overview of the composition and function of the intestinal microbiota and discuss the mechanisms by which the intestinal flora directly or indirectly affects the occurrence and development of gastrointestinal tumours by regulating the immune system, producing bacterial toxins, secreting metabolites. Secondly, we present a detailed analysis of the differences of intestinal microbiota and its pathogenic mechanisms in colorectal cancer, gastric cancer, hepatocellular carcinoma, etc. Lastly, in terms of treatment strategies, we discuss the effects of the intestinal microbiota on the efficacy and toxic side effects of chemotherapy and immunotherapy and address the role of probiotics, prebiotics, FMT and antibiotic in the treatment of gastrointestinal tumours. In summary, this article provides a comprehensive review of the pathogenic mechanisms of and treatment strategies pertaining to the intestinal microbiota in patients with gastrointestinal tumours. And provide a more comprehensive and precise scientific basis for the development of microbiota-based treatments for gastrointestinal tumours and the prevention of such tumours.

## Introduction

Gastrointestinal tumours are common malignant tumours worldwide. Hepatocellular carcinoma (HCC) (8.3%) has the highest mortality rate among gastrointestinal tumours, followed by gastric cancer (GC) (7.7%), colorectal cancer (CRC) (5.8%), and oesophageal cancer (EC) (5.5%), etc. At the same time, colorectal cancer has the highest incidence rate (6.0%) among gastrointestinal tumours, followed by gastric cancer (5.6%), hepatocellular carcinoma (4.7%), and oesophageal cancer (3.1%), etc. [1]. Increasing evidence suggests that gastrointestinal cancers are caused by multiple factors, such as immune system disorders, heavy alcohol consumption, smoking, obesity, diet, environmental factors, and intestinal microbiota dysbiosis [2]. The intestines are a complex environment in which bacteria, fungi, and viruses coexist. The total number of microorganisms in the intestines may be as high as 100 trillion, approximately 10 times the number of human cells in the entire body [3]. The intestinal microbiota plays a major role in the maintenance of human health and the development of diseases. An increasing number of studies have shown that the intestinal microbiota is involved in regulating intestinal function, protecting the intestinal mucosa from pathogens within the intestines, and preventing pathogenic bacteria from escaping from the intestines into other tissues [4] The intestinal microbiota is also closely related to many diseases such as metabolic syndrome [5], depression [6], and tumours [7]. As 16S rDNA sequencing and metagenomics technology rapidly develop, accumulating evidence suggests that the intestinal microbiota is closely related to gastrointestinal tumours. Dysbiosis of the intestinal microbiota leads to increased inflammation and activation of carcinogenic pathways, thereby promoting tumour development. Additionally, metabolites produced by the intestinal microbiota, such as short-chain fatty acids (SCFAs) and bile acids, can regulate the proliferation, apoptosis, and immune functions of gastrointestinal tumour cells, thus influencing tumorigenesis. Furthermore, the intestinal microbiota can enhance the efficacy of chemotherapy and immunotherapy, while also reducing their toxic side effects [8, 9].

In this review, we have summarized the latest literatures and comprehensively explored the pathogenic mechanisms of the gut microbiota and its metabolites in gastrointestinal tumours. This provides a more comprehensive and precise scientific basis for the development of microbiota-based tumours therapies and personalized tumours treatment strategies. Firstly, we introduce the composition and biological functions of the gut microbiota, and discuss the impact of gut microbiota on the oncogenesis and development of gastrointestinal tumours through metabolic pathways, immune pathways, and secretion of protein toxins. We then systematically describe the effects of the gut microbiota and its metabolites on digestive system tumours such as colorectal cancer, gastric cancer, liver cancer, and oesophageal cancer, in order to have a comprehensive understanding of the pathogenic mechanisms. At last, we discuss the effects of the intestinal microbiota on the efficacy and toxic side effects of chemotherapy and immunotherapy and address the role of probiotics, prebiotics, FMT and antibiotic in the treatment of gastrointestinal tumours.

### Composition and function of the intestinal microbiota

#### Composition of the intestinal microbiota

The intestinal microbiota refers to the collection of various microorganisms that inhabit the human intestine, and is primarily composed of bacteria, fungi, viruses, and other microorganisms [3]. Bacteria are the major members of the intestinal microbiota, with the predominant phyla being *Firmicutes*, *Bacteroidetes*, *Actinobacteria*, and *Proteobacteria*, and the predominant genera including *Bacteroides*, *Bifidobacterium*, *Clostridium*, *Lactobacillus*, *Prevotella*, *Ruminococcus*, *Streptococcu*, among others. These bacteria account for more than 90% of the total intestinal microbiota [10]. Based on their functions, the various bacteria within the intestinal microbiota can be categorised as probiotic species, opportunistic pathogens, and pathogenic bacteria. Probiotics beneficial microorganisms that contribute to host health, and include *Bifidobacterium*, *Lactobacillus*, *Butyricicoccus*, *Roseburia*, and more. They facilitate food digestion to produce SCFAs, synthesise vitamins, and enhance immune function, thus playing a crucial role in maintaining gut health [11]. Opportunistic pathogens primarily include *Escherichia coli*, *Streptococcus* (*Enterococcus*), and *Veillonella*. Under normal circumstances, opportunistic pathogens are harmless to their human hosts. However, when immune defences are compromised, or other factors lead to their overgrowth, they can cause intestinal infections, inflammation, food poisoning, and other conditions by secreting toxins [12]. Although the majority of the intestinal microbiota consists of probiotic species and opportunistic pathogens, approximately 10% of the intestinal microbiota comprises pathogenic bacteria, including *Proteus*, *Salmonella*, and *Clostridium difficile*. These pathogenic bacteria can promote the development of gastrointestinal diseases and tumours by secreting harmful metabolites and toxins, disrupting the intestinal mucosal barrier, and inhibiting immune cell function [13, 14]. In summary, maintaining a balanced intestinal microbiota is crucial for preventing intestinal-related diseases and maintaining optimal health.

#### Function of the intestinal microbiota

The intestinal microbiota is an important component of the complex human microecosystem and plays a vital role in regulating metabolism, protecting the intestinal barrier, and modulating the immune system [3, 15]. Studies have shown that bacteria such as *Roseburia*, *Eubacterium rectale*, *Faecalibacterium prausnitzii*, *Clostridium*, and *Butyricicoccus* can metabolise indigestible carbohydrates, such as cellulose, hemicellulose, resistant starch, pectin, oligosaccharides, and lignin, into SCFAs [16]. The SCFAs mainly include acetate, propionate and butyrate. Butyrate is the main energy source for colon epithelial cells and can inhibit over-proliferation of CRC cells, *Escherichia coli*, *Salmonella*, and other pathogens, thus suppressing the development of CRC [17, 18]. The intestinal microbiota also plays a role in vitamin synthesis. *Lactic acid bacteria*, *Roseburia*, and *Bifidobacterium* synthesise vitamins such as biotin, thiamine, cobalamin, riboflavin, vitamin B, and K [19].

The intestinal barrier is a physical barrier that lines the intestinal mucosa and prevents the entry of harmful substances and microorganisms, thereby maintaining intestinal homeostasis [20]. Beneficial bacteria within the intestinal microbiota localise to the surface of the intestinal tract, forming a physical barrier that prevents the attachment and proliferation of harmful bacteria. This competitive inhibition mechanism reduces the abundance of harmful bacteria and diminishes the damage that they could cause to the intestinal mucosa [21]. Some probiotics produce antimicrobial substances such as antimicrobial peptides and organic acids that can directly kill harmful bacteria and prevent them from invading the intestinal mucosa [22]. The intestinal microbiota helps maintain normal function of the intestinal mucosa by producing SCFAs, which promote intestinal cell proliferation and repair [20]. Additionally, the intestinal microbiota can influence the production of adhesive proteins and mucus by mucosal cells, thereby strengthening the protective layer of the intestinal barrier [23].

The intestinal microbiota plays a critical role in the development of the host immune system. Studies have shown that the genus *Clostridium* can promote the generation of regulatory T (Treg) cells in the gut, inhibit overactivation of the immune system, and maintain immune balance [24, 25]. Chuang et al. demonstrated that germ-free mice exhibited decreased immunity, as well as a significant decrease in the numbers of plasma cells and T cells and IgA levels in the intestine. Moreover, colonisation with *segmented filamentous bacteria* can restore the number of T cells in the intestine of germ-free mice [26]. However, the intestinal microbiota is not static, and dysbiosis of the intestinal microbiota due to disruption of homeostasis within the host's internal environment or a decline in immune defences is closely associated with the occurrence and development of gastrointestinal inflammation, mental and psychological diseases, metabolic diseases and malignancies [27–29]. Increasing evidence suggest that the intestinal flora and its products are closely related to gastrointestinal tumours [30–32]. The intestinal microbiota can damage host cells and the intestinal mucosa and influence tumour occurrence, development, and prognosis through secretion of metabolites, modulation of immune cell function, and production of toxins [33–35].

### The effects of the intestinal microbiota and its products on gastrointestinal tumours

In recent years, there has been increasing interest in the role of the intestinal microbiota and its products in the occurrence and development of gastrointestinal tumours. The production of metabolites such as bile acids, trimethylamine oxide (TMAO) and SCFAs by the intestinal microbiota promotes the development of gastrointestinal tumours. Additionally, the intestinal microbiota and its products can influence the occurrence and development of gastrointestinal tumours through immune pathways, and toxin secretion pathways [30, 32, 34, 36, 37]. Therefore, in-depth research on the effects of the intestinal microbiota and its products on gastrointestinal tumours, as well as the underlying mechanisms, is of great significance for the prevention, diagnosis, and treatment of such tumours.

### Metabolite pathways

**Fig. 1:**
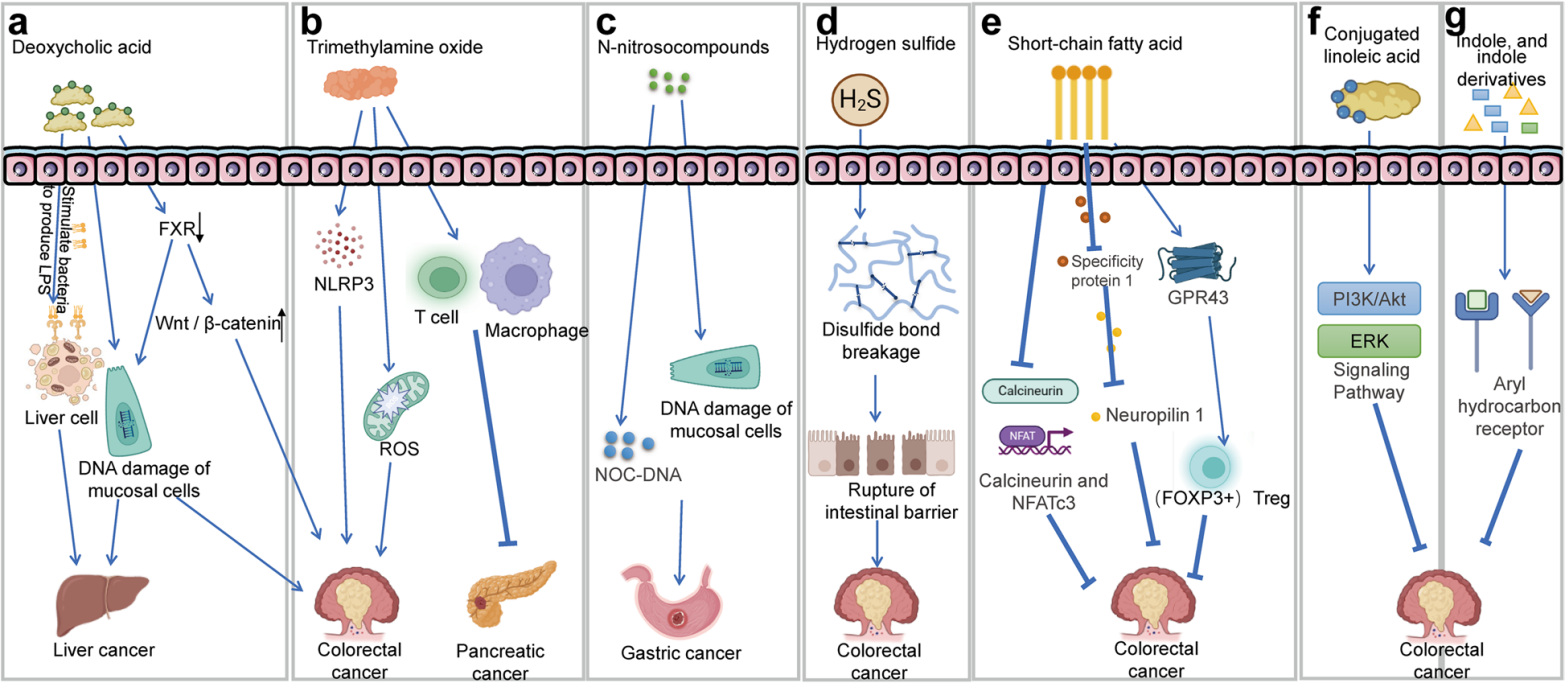
Metabolite pathways. Abbreviations: *LPS* Lipopolysaccharides, *TLR4* Toll-like receptor 4, *FXR* Farnesoid X receptor, *DCA* Deoxycholic acid, *NFATc3* Nuclear factor of activated T cells 3, *FOXP3* Forkhead box P3, *GPCR* G protein-coupled receptor, *ERK* Extracellular signal-regulated kinase, *PI3K/Akt* Phosphatidylinositol 3-kinase/Protein kinase B, *IL* Interleukin

The interaction between the intestinal microbiota and gastrointestinal tumours is mainly mediated by metabolites. Studying the relationship between intestinal microbiota metabolites and gastrointestinal tumours helps reveal the mechanisms by which the intestinal microbiota regulates gastrointestinal tumour development (Fig. 1, Table 1). Pathogenic gut bacteria promote the occurrence and development of gastrointestinal tumours by producing harmful metabolites such as bile acids, TMAO, N-nitrosocompounds (NOCs), and hydrogen sulphide (H_2_S), which damage normal cellular DNA, activate intracellular tumorigenic signalling pathways, and promote the release of inflammatory factors [35]. However, beneficial intestinal bacteria inhibit the occurrence and development of gastrointestinal tumours through the production of beneficial metabolites such as SCFAs, conjugated linoleic acid (CLA), indole, and indole derivatives, which suppress tumorigenic signalling pathways, inhibit the release of inflammatory factors, and decrease the proliferation of pathogenic bacteria and cancer cells.

**Table 1:** Pathogenic mechanism of intestinal microbiota metabolites in gastrointestinal tumours

Metabolite type	Related mechanisms	Reference
Dichloroacetic acid (DCA)	DCA induces DNA damage in liver cells and intestinal mucosal cells. Additionally, DCA reduces the activation of functional farnesoid X receptor (FXR) signalling in CRC cells, thereby promoting the development of colon cancer	[38, 39]
DCA	DCA stimulates Gram-negative bacteria to produce more lipopolysaccharides (LPS), which enter the liver, thereby activating the Toll-like receptor 4 (TLR4) pathway and promoting the occurrence and development of liver cancer	[40]
Trimethylamine oxide (TMAO)	TMAO promotes vascular inflammation by activating the NOD-like receptor family, pyrin domain containing 3 (NLRP3) inflammasome. Additionally, TMAO can stimulate the production of reactive oxygen species (ROS) in the mitochondria	[41, 42]
TMAO	Delivery of TMAO intraperitoneally or via a dietary choline supplement to orthotopic pancreatic cancer bearing mice reduced the tumour growth and associated with an immunostimulatory tumour-associated macrophage phenotype and activated effector T cell response in the tumour microenvironment	[43]
N-nitrosocompounds (NOCs)	NOCs can damage the DNA of mucosal cells, simultaneously generating NOC-DNA adducts that impair the cell's self-repair mechanisms. This disruption leads to dysregulation of cell proliferation and differentiation, ultimately resulting in carcinogenesis	[44–46]
Hydrogen sulfide(H_2_S)	H_2_S can induce DNA damage in normal cells while reducing disulfide bonds in the intestinal epithelial mucus network. This ultimately leads to the breakdown of the intestinal mucosal barrier, exposing the intestinal epithelium to bacteria and toxins, resulting in inflammation	[47, 48]
Short-chain fatty acids (SCFAs)	SCFAs can inhibit the excessive proliferation of colorectal cancer cells and gut pathogenic bacteria such as Escherichia coli and Salmonella	[18]
SCFAs	SCFAs help inhibit the proliferation of colorectal tumour cells by inhibiting the activation of calcineurin and nuclear factor 3 (NFATc3) of activated T cells	[49]
SCFAs	Butyrate-mediated activation of GPR109A could upregulate anti-inflammatory effector molecules IL-10 and Aldh1a in colonic DCs and macrophages, which promoted the differentiation of IL-10-producing CD4 T cells and Tregs, and inhibited the development of IL17-producing T cells, thereby inhibiting the occurrence of CRC	[50]
SCFAs	Acetate and propionate could induce forkhead box P3 (FOXP3 +) Tregs in a GPR43-dependent manner to protect CRC	[51, 52]
Conjugated linoleic acid (CLA)	CLA helps inhibit the PI3K/Akt and ERK signalling cascades, inducing apoptosis in tumour cells	[53]
Indole, and indole derivatives	Indole, and indole derivatives are the major ligands for the aryl hydrocarbon receptor (AhR). They can activate AhR, thereby maintaining intestinal homeostasis, inhibiting pathogen infection, and improving symptoms of colitis	[54, 55]

Abbreviations: *CRC* Colorectal cancer, *TNF* Tumour necrosis factor, *NF-κB* Nuclear factor-kappa B, *ERK* Extracellular signal-regulated kinase, *PI3K/Akt* Phosphatidylinositol 3-kinase/Protein kinase B, *TLR4* Toll-like receptor 4, *IL* Interleukin, *TGF-β* Transforming Growth Factor-beta, *Bcl-2* B-cell lymphoma 2

Intestinal microbiota (e.g., *Bacteroides fragilis*, *Bacteroides vulgatus*, *Clostridium perfringens*, *Eubacterium*, *Lactobacillus*, and *Bifidobacterium*) metabolize primary bile acids to secondary bile acids by 7α-dehydroxylation [35]. After entering the liver through the portal vein, deoxycholic acid (DCA), a derivative of secondary bile acids, induces DNA damage in liver cells and intestinal mucosal cells [56]. DCA also dissolves epithelial tissue to stimulate bacteria to produce more lipopolysaccharide (LPS) that enters the liver, thereby activating the Toll-like receptor 4 (TLR4) pathway and promoting the development of HCC [40]. Mechanistically, DCA promotes the growth of CRC cells by inducing the activation of the epidermal growth factor receptor (EGFR) and Wingless/integrated (Wnt) signalling pathways [57, 58]. Additionally, in CRC, DCA reduces activation of functional farnesoid X receptor (FXR) signalling in CRC cells, thereby promoting the occurrence of CRC [38, 39]. Recent studies have shown that a lack of FXR not only impairs intestinal and hepatic circulation and bile acid production but also enhances the canonical Wnt/β-catenin signalling pathways, thereby promoting DNA damage [35, 59].

TMAO is a metabolite of trimethylamine (TMA), which is primarily produced by *Clostridium anaerobes* (*phylum Clostridia*) and *Facultative Anaerobic Enterobacteriaceae* (*phylum Proteobacteria*) during the digestion of foods rich in choline and carnitine, such as meat, eggs, and fish [60, 61]. Although there is no direct evidence suggesting that TMAO directly promotes the occurrence and development of gastrointestinal tumours, numerous studies have shown that TMAO can induce inflammation and oxidative stress. TMAO promotes vascular inflammation by activating the NOD-like receptor family, pyrin domain containing 3 (NLRP3) inflammasome and can also stimulate reactive oxygen species (ROS) production in mitochondria [41, 42]. Clinical studies have shown that plasma TMAO levels are positively correlated with CRC [62]. However, Gauri et al. found that TMAO enhances antitumour immunity in pancreatic cancer (PC). Delivery of TMAO to orthotopic PC–bearing mice intraperitoneally or via a dietary choline supplement reduced tumour growth and was associated with an immunostimulatory tumour-associated macrophage phenotype and activated effector T cell response in the tumour microenvironment (TME) [43]. However, the precise mechanism by which TMAO promotes tumorigenesis is still unclear and requires further exploration.

NOCs are a group of compounds containing nitroso groups, including N-nitrosamines and N-nitrosamides. NOCs are potent carcinogens and have been associated with the occurrence and development of gastrointestinal tumours, such as GC and CRC. In the gastrointestinal tract, nitrite undergoes nitrosation to form NOCs. The high concentration of NOCs can damage colon mucosal cell DNA and generate NOC-DNA adducts, resulting in loss of the self-repair ability in damaged cells, leading to cell proliferation and differentiation disorders, and ultimately inducing carcinogenesis [44–46].

Sulphate-reducing bacteria (SRB) generate H_2_S by metabolising sulphate in food and other sulphur-containing compounds, such as taurine and cysteine [63, 64]. Extensive research has shown that the levels of H_2_S in the faeces of gastrointestinal tumour patients are significantly higher than those found in the faeces of healthy individuals, indicating that H_2_S plays a role in the pathogenesis of gastrointestinal tumours [65, 66]. H_2_S has been found to reduce disulfide bonds in the mucin network of intestinal epithelium, leading to disruption of the intestinal mucosal barrier and exposing the intestinal epithelium to bacteria and toxins, ultimately triggering inflammation [47]. Additionally, Matias et al. demonstrated that H_2_S can induce DNA damage in normal cells, in a process that may be mediated by free radicals [48]. Interestingly, some studies have suggested that H_2_S can protect and rebuild the damaged mucus layer, thereby preventing inflammation [67, 68]. Further research is needed to explore the mechanisms by which H_2_S affects the development of CRC, as it remains controversial whether H_2_S acts as a promoting or inhibiting factor.

Beneficial metabolites SCFAs are a type of organic acid produced by the metabolism of dietary fibre and undigested carbohydrates by the intestinal microbiota, mainly including acetic acid, propionic acid, and butyric acid [69]. These SCFAs are primarily produced by *Bacteroides*, *Coprococcus comes*, *Anaerostipes spp*, *Eubacterium rectale*, *Faecalibacterium prausnitzii*, and *Roseburia spp.*[70]. SCFAs inhibit the development of gastrointestinal tumours through a variety of mechanisms, such as inhibiting the proliferation of pathogenic bacteria and cancer cells, inducing cancer cell apoptosis, and suppressing inflammatory responses. Research has shown that SCFAs can inhibit excessive proliferation of pathogenic intestinal bacteria and the growth of CRC cells [18]. SCFAs serve as the main energy source for colon cells, promote the proliferation of colon epithelial cells, protect the intestinal mucosa, and help prevent CRC [71]. SCFAs help inhibit protein acetylation and inhibit the proliferation of CRC cells by inhibiting activation of calcineurin and nuclear factor of activated T cells 3 (NFATc3) [49]. Butyrate could diminish the expression of neuropilin 1 by suppressing the transactivation of Specificity protein 1 (Sp1) to inhibit the angiogenesis, metastasis, and survival of CRC cells, and butyrate could trigger the CRC cells apoptosis by activating the signalling of Wnt [51]. Activation of G protein-coupled receptors (GPCRs) inhibits the development of colonic inflammation and CRC, while SCFAs can act as ligands for certain GPCRs. There are three main types of GPCRs associated with SCFAs: GPR109A, GPR43 and GPR41 [72]. Mechanistically, Singh et al. found that butyrate-mediated activation of GPR109A could upregulate anti-inflammatory effector molecules IL-10 and Aldh1a in colonic DCs and macrophages, which promoted the differentiation of IL-10-producing CD4 T cells and Tregs, and inhibited the development of IL17-producing T cells, thereby inhibiting the occurrence of CRC [50]. Acetate and propionate could induce forkhead box P3 (FOXP3 +) Tregs in a GPR43-dependent manner to protect CRC [51, 52]. In addition, SCFAs promote IL-22 production by CD4 T cells and ILCs through GPR41 and inhibition of histone deacetylase, thereby alleviating colorectal inflammation [73].. Additionally, Kobayashi et al. showed that in SCFAs enhance the cytotoxicity of cisplatin against HCC cells by regulating the G protein-coupled receptor 41 signalling pathway [74].

In addition to SCFAs, beneficial metabolites such as CLA, indole, and indole derivatives can also inhibit the occurrence and development of gastrointestinal tumours. CLA is an isomer of LA, and linoleic acid can be converted into CLA by *Lactobacilli* and *Bifidobacterium*. Research has shown that CLA can inhibit tumour cell apoptosis and proliferation [75–77]. Mechanistically, CLA can suppress the cascade connection of Phosphatidylinositol 3-kinase/Protein kinase B (PI3K/Akt) and Extracellular signal-regulated kinase (ERK) signalling pathway, induce cell apoptosis, and inhibit the cell cycle in cancer cell lines [53]. Trans-10, cis-12 CLA induces colon cancer cell apoptosis by inducing ROS production and endoplasmic reticulum stress [76]. Indole and its derivatives are produced by *Bacteroides*, *Enterococcus*, and *Lactobacillus* via tryptophan metabolism [78]. Studies have shown that indole and its derivatives are major ligands for aryl hydrocarbon receptor (AhR) that induce AhR activation, thereby maintaining intestinal homeostasis, inhibiting pathogen infection, and improving colitis symptoms [54, 55].

In summary, metabolites mediate the interaction between the intestinal microbiota and gastrointestinal tumours. Pathogenic bacteria damage normal cellular DNA, activate oncogenic signalling pathways, and promote the release of pro-inflammatory factors by producing harmful metabolites such as TMAO, NOCs, and H_2_S, thereby driving tumour development. Probiotics produce beneficial metabolites such as SCFAs, CLA, and indole that inhibit cancer signalling pathways, reduce the release of inflammatory factors, and suppress the growth of pathogenic bacteria and cancer cells, thereby inhibiting tumour development. Therefore, further research on the relationship among metabolites, the intestinal microbiota, and tumour development is expected to provide new insights into the treatment of gastrointestinal tumours.

### Immune pathway

The TME is a specialized and complex biological environment that affects the growth, invasion, and metastasis of tumour cells. Many types of immune cells are present in the TME, including T cells, B cells, macrophages, natural killer (NK) cells, adipocytes, fibroblasts, and myeloid-derived suppressor cells (MDSCs). However, increasing evidence suggests that microorganisms are also major components of certain TMEs [79]. The gut microbiome, as an important participant in the gut environment, is involved in immune regulation and related to the tumour immune response [80, 81]. Moreover, the gut microbiome imbalance may promote or inhibit tumour development by reshaping the TME and altering the immune response [33].

**Fig. 2:**
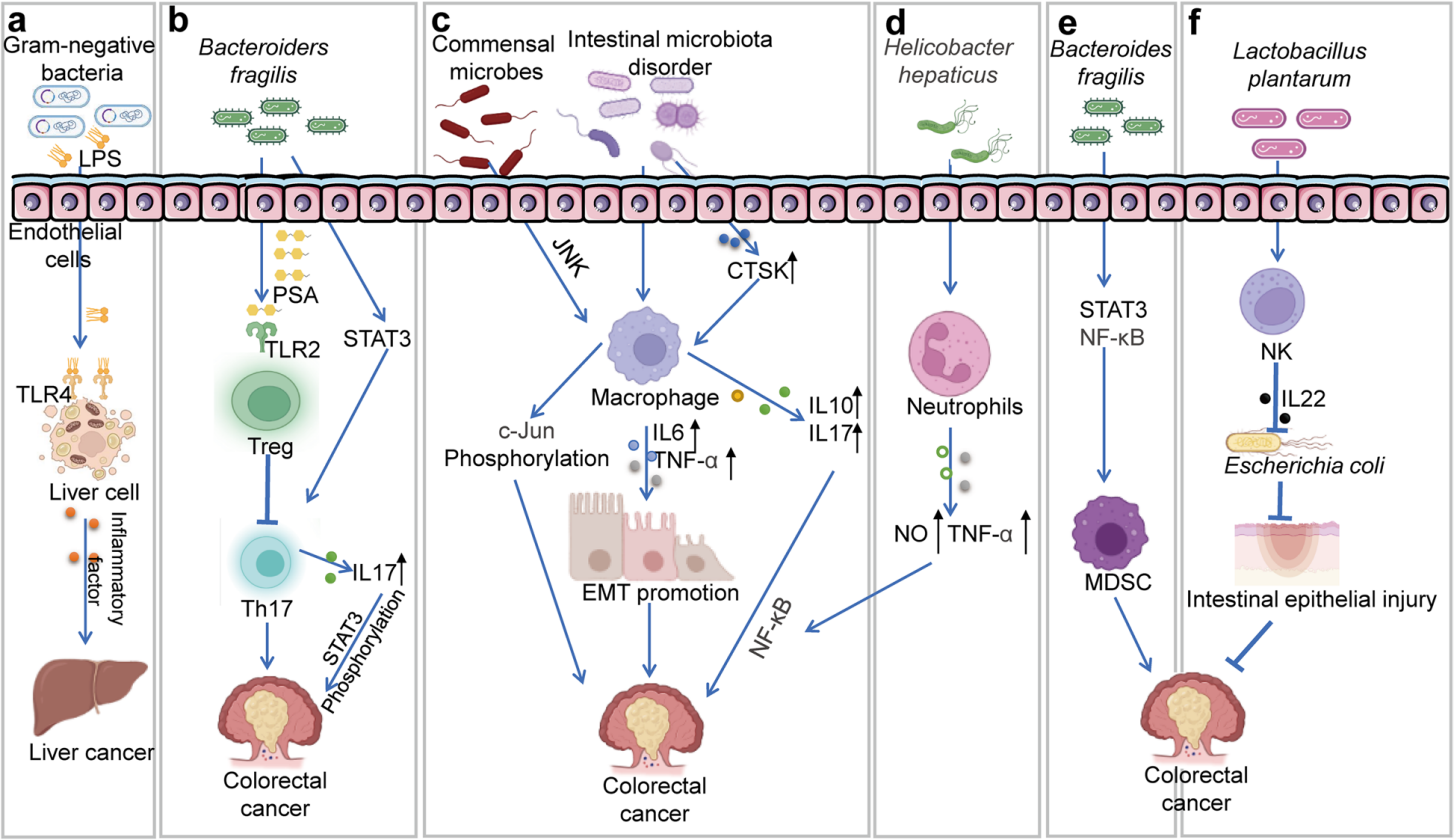
Immune pathways. Abbreviations: *LPS* Lipopolysaccharides, *PSA* Polysaccharide A, *TNF* Tumour necrosis factor, *NF-κB* Nuclear factor-kappa B, *FadA*, *Fusobacterium Nucleatum* Adhesin A, *BFT Bacteroides fragilis* toxin, *TLR2/4* Toll-like receptor 2/4, *Treg* Regulatory T cell, *IL-10/17/6/22* Interleukin-10/17/6/22, *Th17* Helper T cell 17, *STAT3* Signal transduction and transcription factor 3, *EMT* Epithelial-mesenchymal transition, *CTSK* Cathepsin K, *SAPK* Stress-activated protein kinase, *NO* Nitric oxide, *MDSC* Myeloid-derived suppressor cell, *NK* Natural killer cell

The gut microbiome has a bidirectional regulatory effect on the TME. The gut microbiome affects the occurrence and development of tumours by regulating the functions of various immune cell types (Fig. 2). LPS is a component of the cell wall of gram-negative bacteria in the gut and binds to TLR4 on the surface of liver cells, activating the NF-κB signalling pathway and promoting immune inflammatory reactions, leading to the release of a large number of pro-inflammatory cytokines that promote the occurrence and development of HCC [82]. Liu et al. demonstrated that TLR2 mRNA and protein expression are significantly upregulated in colorectal tumours. Furthermore, TLR2 promotes CRC cell proliferation by activating the PI3K/Akt and NF-κB signal pathways [83]. Additionally, in the gastrointestinal tract, LPS stimulates intestinal epithelial cells through the TLR pathway, induces phosphorylation of IL-1 receptor-associated kinase and mitogen-activated protein kinase (MAPK), promotes IL-8 expression, and induces inflammation [84]. Additionally, enterotoxigenic *Bacteroides fragilis* activates Th17 cells through the signal transduction and transcription factor 3 (STAT3) signalling pathway. Th17 cells release IL-17, which promotes STAT3 phosphorylation in tumour cells, thereby promoting tumour development [85]. *Lactobacillus rhamnosus* increases Treg activation, and Tregs suppress the occurrence and development of breast tumours by promoting apoptosis [86]. These findings suggest that some intestinal microbiota affect tumour development by regulating the function and quantity of T cell subsets.

Intestinal microbiota also influences the occurrence and development of tumours by affecting tumour-associated macrophages (TAMs) in the TME. Wan et al. found that an intestinal microbiota imbalance leads to TAM activation, which subsequently promotes the secretion of IL-6 and TNF-α. IL-6 and TNF-α accelerate the development of CRC by promoting epithelial–mesenchymal transition (EMT) formation [87]. The intestinal microbiota imbalance also induces secretion of cathepsin K by tumour cells, which activates macrophages through the mammalian target of rapamycin pathway. Cathepsin K stimulates macrophages to secrete IL-10 and IL-17, which promote CRC cell invasion and metastasis through the NF-κB signalling pathway [88]. Additionally, some commensal microbiota stimulates macrophages to promote CRC cell proliferation by activating the stress-activated protein kinase signalling pathway and increasing c-Jun phosphorylation in CRC cells [89].

In addition to macrophages and T cells, intestinal microbiota influences the occurrence and development of tumours through neutrophils, NK cells, and MDSCs. Studies have shown that *Helicobacter hepaticus* activates neutrophils, which increase the levels of nitric oxide (NO) and TNF-α, and promote CRC development by activating the NF-κB signalling pathway [90]. Erik et al. reported that *Bacteroides fragilis* directly promote the occurrence of colon tumours by activating MDSCs through STAT3 and NF-κB signalling pathways [85]. Takuya et al. showed that *Lactobacillus plantarum*-induced NK cells secrete IL-22, which protects against enterotoxigenic *Escherichia coli*-induced intestinal epithelial barrier damage [91]. In summary, certain intestinal microbiota promotes or inhibits the occurrence of tumours by affecting various immune cells in the TME.

### Bacterial toxin pathway

**Fig. 3:**
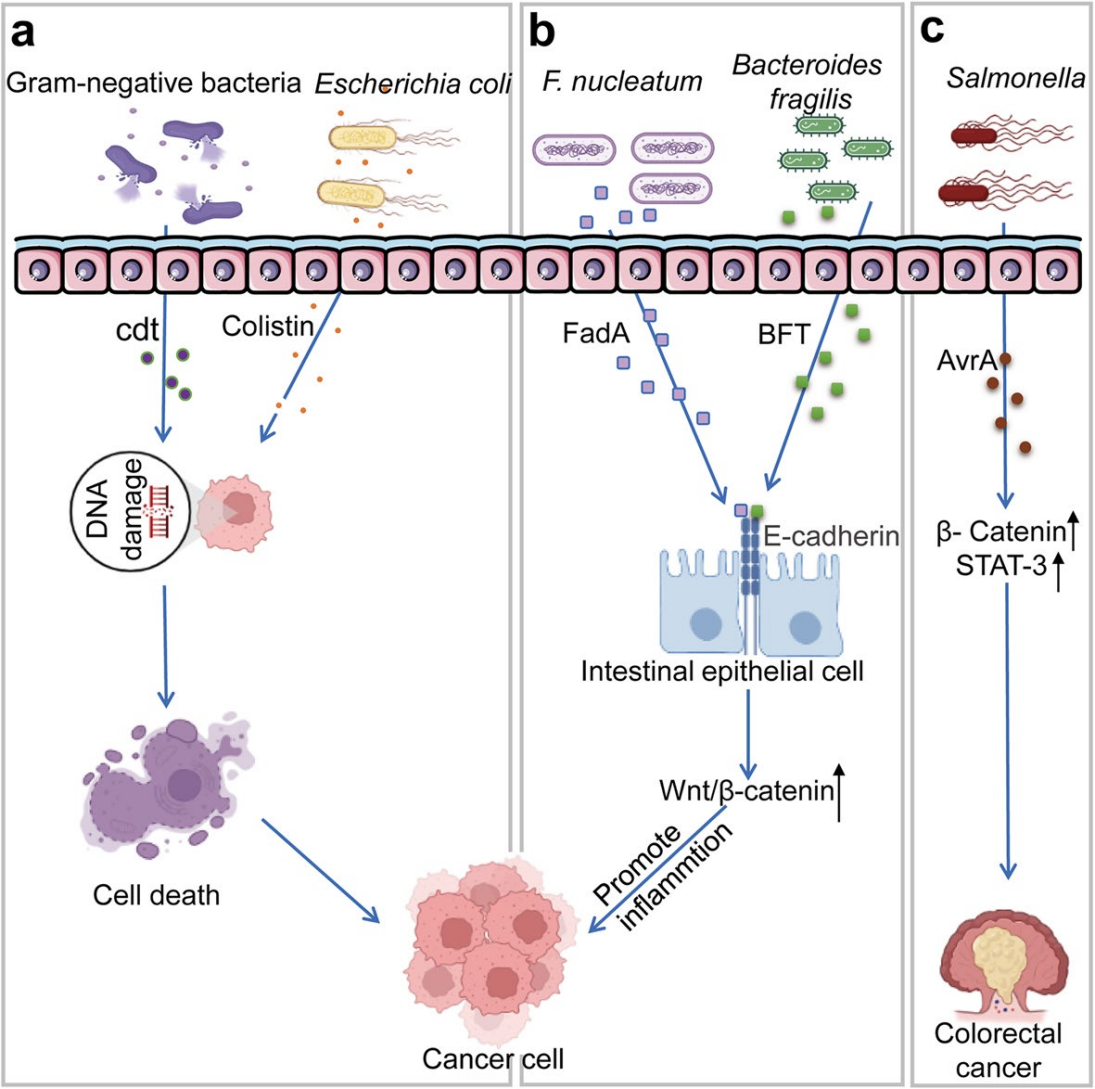
Bacterial toxin pathways. Abbreviations: *Cdt* Cytolethal distending toxin, *FadA Fusobacterium nucleatum* adhesin A, *BFT* Bacteroides fragilis toxin, *AvrA* Avirulence protein A, *STAT3* Signal transduction and transcription factor 3

Increasing evidence suggests that pathogenic bacteria induce host cell DNA damage by producing protein toxins or inducing inflammation through interference of apoptosis and cell proliferation, thereby promoting tumour development (Fig. 3) [34]. Cytotoxic dilatoxin (Cdts) are a group of heat-labile protein exotoxins secreted by more than 30 pathogenic gram-negative bacteria [92]. Cdt enters cells through endocytosis, and exerts its cytotoxic effects in the nucleus [93]. Moreover, Cdt induces low levels of single-stranded DNA breaks and high levels of double-stranded DNA breaks (DSBs) in host cells [94]. When the degree of DNA damage exceeds the ability of host cells to repair, cell death or carcinogenic mutations occur. In addition to Cdt, colibactin secreted by B2-group *Escherichia coli* is also associated with DSB formation [95]. *Escherichia coli* promotes crosslinking between DDR, single-stranded DNA breaks, and DNA in eukaryotic cells by inducing DSBs [96], which affects the cell cycle and leads to apoptosis or senescence [97].

In addition to causing DNA damage, bacterial genotoxins regulate signalling pathways, thereby promoting tumour initiation and progression. A bacterial cell surface adhesin expressed by *Fusobacterium nucleatum* known as *Fusobacterium nucleatum* adhesin A (FadA), and *Bacteroides fragilis* toxin secreted by enterotoxigenic *Bacteroides fragilis*, when combined with host intestinal epithelial cell E-cadherin, regulate the canonical Wnt/β-catenin signalling pathway. As a result, the expression of Wnt pathway-related transcription factors, oncogenes, and inflammation-related genes increases, leading to increased intestinal epithelial cell proliferation, barrier disruption, and promotion of mucosal inflammation and carcinogenesis [13, 98]. Lu et al. showed that avirulence protein A (AvrA), a bacterial effector protein produced by *Salmonella* strains, promotes tumorigenesis through sustained activation of β-catenin and upregulation of STAT-3 [99, 100].

### Imbalances in the intestinal microbiota and the occurrence and development of gastrointestinal tumours

Research has shown that dysbiosis of the intestinal microbiota is closely associated with the occurrence and development of gastrointestinal tumours such as CRC, HCC and GC. The intestinal microbiota maintains intestinal stability and health. However, when the intestinal microbiota becomes imbalanced, as characterised by changes in composition or abnormal fluctuations in quantity (Table 2), these changes may increase the risk of developing gastrointestinal tumours. An imbalance in the intestinal microbiota may promote the occurrence and development of gastrointestinal tumours through secretion of toxic metabolites, regulation of immune responses, and secretion of protein toxins (Table 3).

**Table 2:** Abundance differences and prognosis of different bacterial communities in gastrointestinal tumours

Type	Intestinal microbiota	Reference
Colorectal cancer	In the gut of colorectal cancer patients, the abundance of *Oral Peptostreptococcus, Proteus, Fusobacterium nucleatum, Escherichia coli fragilis* and *Enterococcus faecalis* was high, while the abundance of *Rothia, Clostridium, Faecal Bacillus* and *Bifidobacterium* was generally reduced	[101, 102]
Colorectal cancer	Significant enrichment of *Fusobacterium nucleatum* could serve as a biomarker for colorectal cancer (AUC: 0.8) and were associated with a poor prognosis	[103]
Colorectal cancer	11 metabolite markers (2-hydroxybutyric acid, gamma-aminobutyric acid, L-alanine, L-aspartic acid, norvaline, ornithine, oxoadipic acid, oxoglutaric acid, palmitoleic acid, phenylacetic acid and pimelic acid) and 6 bacterial species (*Fusobacterium nucleatu, Peptostreptococcus anaerobius, Peptostreptococcus micros, Ruminiclostridium inulinivorans, Eikenella corrodens* and *Xanthomonas. perforin*) achieved a higher AUC of 0.9417	[104]
Colorectal cancer	*Bacillus fragilis* and faecal bacteria were positively correlated with prognosis. *Fusobacterium nucleatum* and *Bacteroides fragilis* were negatively correlated with prognosis.a	[105]
Gastric cancer	The aerobic bacteria (*streptococci* and *enterococci*) and facultative anaerobes (*Escherichia*, *Enterobacter* and *Streptococcus*) in gastric cancer patients increased significantly after gastrectomy because of the increase of intestinal oxygen and the translocation of oral flora	[32]
Gastric cancer	After chemotherapy, the PFS of gastric cancer patients with increased R. faeces abundance was longer	[106]
Hepatocellular Carcinoma	Butyrate-producing bacteria decreased significantly, such as *Ruminococcus*, *Oscillibacter*, *Faecalibacterium*, *Clostridium IV* and *Coprococcus,* while there was an increase in *Klebsiella* and *Haemophilus* that produce Lipopolysaccharides	[107]
Hepatocellular Carcinoma	The abundance of *Blautia* was positively correlated with progression-free survival and overall survival in liver cancer patients undergoing chemotherapy	[108]
Hepatocellular Carcinoma	Significant enrichment of *Bacteroides, Lachnospiracea incertae sedis*, and *Clostridium XIVa* in liver cancer patients was associated with poor clinical prognosis	[109]
Oesophageal cancer	The abundance of *Streptococcus*, *Prevobacter*, *Clostridium*, *Velococcus* and *Lactobacillus* increased in patients with oesophageal cancer	[110]
Oesophageal cancer	*Streptococcus* and *Prevotella* are biomarkers of poor prognosis in oesophageal cancer	[111]
Pancreatic Cancer	Studies have shown that the abundance of *Veillonella atypica, Fusobacterium nucleatum/hwasookii* and *Alloscardovia omnicolens* in pancreatic cancer tissues and stool samples is significantly increased	[112]
Pancreatic Cancer	The poor prognosis of pancreatic cancer was associated with the *Fusobacterium*	[113]

Abbreviations: *AUC* Area under the curve

**Table 3:** Different roles and possible mechanisms of different bacterial communities in gastrointestinal tumours

Type	Main related intestinal microbiota	Related mechanisms	Reference
Colorectal Cancer	Bacteria that produce short chain fatty acids (SCFAs)	SCFAs help inhibit protein acetylation and inhibit the proliferation of CRC cells by inhibiting activation of calcineurin and nuclear factor of activated T cells 3 (NFATc3)	[49]
Colorectal Cancer	*Fusobacterium nucleatum* and enterotoxigenic *Bacteroides fragilis*	*Fusobacterium nucleatum* secretes FadA and enterotoxigenic *Bacteroides fragilis* secretes BFT to regulate the Canonical Wnt/β-catenin signalling pathway, causing intestinal inflammation and damaging the intestinal mucosa, inducing the occurrence of CRC. Additionally, cytotoxic dilation toxins can induce cell DNA damage, interfere with cell cycle and apoptosis	[13, 94, 98]
Colorectal Cancer	Intestinal microbiota imbalance	Dysbiosis of intestinal microbiota leads to activation of tumour-associated macrophages (TAMs), promoting secretion of IL-6 and TNF-α, which accelerates the development of CRC by inducing epithelial-mesenchymal transition (EMT)	[87]
Hepatocellular Carcinoma	Lipopolysaccharides (LPS) in the cell wall of Gramnegative bacteria	LPS binds to TLR4 on liver cells, activating the NF-κB signalling pathway, which in turn activates the immune inflammatory response and promotes the release of a large number of inflammatory factors, ultimately accelerating the development of Hepatocellular Carcinoma	[82]
Hepatocellular Carcinoma	*Clostridium*	Clostridium promote the breakdown of primary bile acids into secondary bile acids. The derivative of secondary bile acid (DCA) induces DNA damage in liver cells and intestinal mucosal cells	[112]
Oesophageal cancer	LPS in the cell wall of Gramnegative bacteria	TLR4 expression is significantly increased in the oesophageal cells of patients with oesophageal cancer. LPS binds to TLR4 and activates the NF-κB pathway, promoting the formation of oesophageal cancer. Meanwhile, LPS directly affects the function of the lower oesophageal sphincter, indirectly promoting the development of oesophageal cancer	[114]
Oesophageal cancer	*Fusobacterium nucleatum*	Infection with *Fusobacterium nucleatum* can induce increased expression of high-mobility group box 1 protein (HMGB1), thus promoting the proliferation of oesophageal cancer cell lines	[115, 116]
Pancreatic Cancer	Microbe-associated molecular patterns (MAMPs) such as lipopolysaccharide and peptidoglycan	The binding of Toll-like receptors to MAMPs and damage-associated molecular patterns (DAMPs) activates the NF-κB and MAPK pathways, resulting in excessive secretion of pro-inflammatory cytokines that promote the occurrence and development of pancreatic cancer	[117–119]

Abbreviations: *CRC* Colorectal cancer, *TNF* Tumour necrosis factor, *NF-κB* Nuclear factor-kappa B, *FadA* *Fusobacterium Nucleatum* Adhesin A, *BFT* *Bacteroides fragilis* toxin, *TLR4* Toll-like receptor 4, *IL* Interleukin, *MAPK* Mitogen-activated protein kinase

### Colorectal cancer

CRC is one of the most common malignant tumours, ranking second in global cancer deaths in 2018 [1]. Compared with healthy individuals, microbiota in CRC patients is primarily composed of pathogenic bacteria associated with metabolic disorders, while the abundance of probiotics, such as butyrate-producing bacteria, is reduced [101]. *Oral Peptostreptococcus, Proteus, Fusobacterium nucleatum, Escherichia coli fragilis*, and *Enterococcus faecalis* are high in CRC patients, while the abundance of *Rothia, Clostridium, Feacal Bacillus*, and *Bifidobacterium* is generally reduced in CRC patients [101, 102]. Combining metabolic products and bacterial markers can more accurately distinguish CRC patients from healthy individuals. Yu et al. established a random forest model by combining 11 metabolites and six bacterial species, obtaining an area under the curve (AUC) of 0.9417 [104].

In CRC, pathogenic bacteria in the gut mainly promote tumour development through three pathways: regulating immune responses, secreting metabolites, and producing bacterial toxins. In terms of the immune pathway, dysbiosis of intestinal microbiota activates TAMs, which promotes the secretion of IL-6 and TNF-α. In turn, IL-6 and TNF-α accelerate CRC development by promoting EMT [87]. *Helicobacter hepaticus* activates neutrophils to produce NO and TNF-α, which activate the NF-κB signalling pathway and promote CRC development [90]. Additionally, *Bacteroides fragilis* directly promotes the development of colorectal tumours through activation of MDSCs via STAT3 and NF-κB signalling pathways [85, 120]. In terms of the genotoxin pathway, *Fusobacterium nucleatum* produces *Fusobacterium nucleatum* adhesin A, while enterotoxigenic *Bacteroides fragilis* secretes *Bacteroides fragilis* toxin. These toxins bind to E-cadherin on intestinal epithelial cells and regulate the canonical Wnt/β-catenin signalling pathway, causing inflammation, disrupting the intestinal mucosa, and inducing CRC [13, 98]. Cdt induces DNA damage in intestinal cells, interferes with the cell cycle, and inhibits apoptosis [94]. In terms of the metabolite pathway, the metabolites produced by intestinal microbiota also influence the onset and development of CRC. Dysbiosis of intestinal microbiota leads to the production of secondary bile acids that cause DNA damage by generating pro-oxidant molecules such as ROS and nitrogen-containing substances [121]. Furthermore, DCA reduces activation of the FXR signalling pathway in CRC cells, thereby promoting tumour development [38, 39]. SCFAs help inhibit protein acetylation and inhibit the proliferation of CRC cells by inhibiting activation of calcineurin and nuclear factor of activated T cells 3 (NFATc3) [49].

### Gastric cancer

GC is one of the most common malignant tumours worldwide, and its mortality ranks fourth [1]. With the development of high-throughput technologies and metagenomic sequencing, and 16S rDNA sequencing, significant differences have been found in intestinal microbiota between GC patients and healthy individuals. After surgery, because of increased oxygen in the gut and translocation of oral microbial communities, there is a significant increase in obligate aerobes (*Streptococc*i and *Enterococci*) and facultative anaerobes (*Escherichia*, *Enterobacter*, and *Streptococcus*) in GC patients [122–124]. The stomach and intestine are directly connected in the human body. The abundance of multiple dominant components of the intestinal microbiota that are associated with CRC, such as *Fusobacterium nucleatum*, *Atopobium parvulum*, and *Escherichia coli*, is significantly increased in post-gastrectomy patients [122]. Moreover, these patients have a twofold increased risk of CRC compared with patients with other types of cancer [125–127]. Post-gastrectomy patients exhibit increased proliferation of the rectal mucosa, and levels of the risk biomarker for CRC, macrophage migration inhibitory factor (MIF), continues to rise within three years after surgery [128]. Additionally, research has demonstrated that dysbiosis of the intestinal microbiota in post-gastrectomy patients mediates changes in certain intestinal metabolites and metabolic pathways, thereby promoting CRC tumorigenesis [122, 129, 130]. For instance, dysbiosis of the intestinal microbiota in GC patients can indirectly promote CRC tumorigenesis and development through increased secondary bile acid production and decreased short-chain fatty acid production [129].Moreover, in the gastrointestinal tract, nitrite undergoes nitrosation to form NOCs, which can damage DNA in mucosal cells. It also can generate NOC-DNA adduct, which makes damaged cells lose the ability of DNA self-repair, leading to cellular proliferation and differentiation disorders, creating an inflammatory environment and ultimately inducing carcinogenesis [44].

### Hepatocellular carcinoma

HCC is the third leading cause of cancer death [1]. Compared with healthy individuals, HCC patients show a significant decrease in bacteria that produce butyrate, such as *Ruminococcus, Oscillibacter, Faecalibacterium, Clostridium IV,* and *Coprococcus*, while bacteria that produce LPS, such as *Klebsiella* and *Haemophilus*, increase.

The gut and liver are anatomically and physiologically connected, and thus the relationship between the gut and liver is referred to as the gut–liver axis [37]. Dysbiosis of intestinal microbiota can lead to intestinal mucosal damage. Large amounts of bacterial metabolites and intestinal microbiota itself can be transferred to the liver through the portal vein, inducing inflammation and promoting the occurrence and development of HCC [131, 132]. LPS, also known as endotoxin, is a complex polysaccharide molecule that is present on the outer membrane of bacteria. It is primarily found in gram-negative bacteria, such as *Escherichia coli* and *Salmonella* [133]. Moreover, when intestinal microbiota dysbiosis occurs, LPS can be translocated to the liver. LPS translocation directly recognizes TLR4 on liver cells and activates NF-κB, leading to enhanced tumour cell proliferation [134]. Additionally, intestinal microbiota dysbiosis promotes the metabolism of primary bile acids into secondary bile acids, such as DCA and lithocholic acid (LCA), which cause liver cell damage and promote HCC [135]. Jang et al. demonstrated that intestinal microbiota, such as *Clostridium*, promote the breakdown of primary bile acids into secondary bile acids. The derivative of DCA induces DNA damage in liver cells and intestinal mucosal cells, thereby promoting the development of HCC [136]. The liver serves as the metabolic centre for choline, which aids in lipid transport within liver cells and prevents abnormal lipid accumulation in the liver. Choline deficiency is often associated with liver steatosis [37]. *Firmicutes* and *Proteobacteria* are capable of metabolizing choline into TMA, which is then transported to the liver and converted into TMAO[60, 61]. TMAO can induce inflammatory responses and oxidative stress, thereby promoting the development of HCC [41, 42, 137]. In summary, the intestinal microbiota promotes the occurrence and progression of HCC through various pathways such as intestinal mucosal damage, LPS translocation, bile acid conversion, and abnormal choline metabolism.

### Oesophageal cancer

EC is the eighth most common type of cancer worldwide and the sixth leading cause of cancer deaths [1]. Microbiota diversity and richness in patients with oesophageal squamous cell carcinoma (ESCC) and postoperative patients are significantly lower than those in healthy individuals, and the abundance of *Streptococcus*, *Prevotella*, *Clostridium*, *Veillonella*, and *Lactobacillus* increases in EC patients. PICRUSt results showed that ESCC patients had mainly enriched pathways related to cysteine, monosaccharide, and starch metabolism, while the levels of fatty acids, SCFAs, tryptophan, and β-alanine metabolism were lower [110]. In EC, intestinal pathogenic bacteria mainly promote tumour occurrence by regulating immune response pathways. In terms of immune pathways, TLR4 increases in oesophageal epithelial cells of oesophageal cancer patients. And due to the dysbiosis of intestinal flora, LPS binds to TLR4 and activates the NF-κB pathway, promoting the formation of oesophageal adenocarcinoma. LPS directly affects the function of the oesophageal lower oesophageal sphincter, indirectly promoting the development of oesophageal adenocarcinoma [114]. Additionally, enrichment of Fusobacterium in EC patients negatively correlates to patient survival [110]. *Fusobacterium* induces the expression of proinflammatory cytokines in epithelial cells, including IL-6 and IL-8, which may contribute to dynamic crosstalk between tumour cells and cancer-associated fibroblasts (CAF) in the ESCC TME [115]. *Fusobacterium* infection also induces the expression of high-mobility group box 1 (HMGB1) protein, which may lead to increased proliferation of ESCC cells [116].

### Pancreatic cancer

PC is currently the seventh leading cause of cancer-related death [1]. The abundance of *Veillonella atypica, Fusobacterium nucleatum/hwasookii*, and *Alloscardovia omnicolens* significantly increases in PC tissues and faecal samples [112]. The detection rate of genus *Clostridium* in PC tissues is 8.8%, and *Clostridium* is associated with a poor prognosis [113, 138]. The liver and pancreas are directly connected to the intestine through the bile duct and pancreatic duct, allowing harmful intestinal microbiota components and metabolites to reach the pancreas via the "intestine-liver-pancreas axis," thereby promoting the development of PC [139]. These findings indicate that intestinal flora in PC patients do not promote the occurrence or development of PC through direct actions, but through chronic low-level activation of the immune system and continuous tumour inflammation, thereby promoting the occurrence of PC [117, 118, 140]. TLRs are a family of pattern recognition receptors (PRR) expressed on the vast majority of immune cells. When intestinal flora is imbalanced, TLRs recognize various microbe-associated molecular patterns (MAMP), such as LPS and peptidoglycans. In addition, TLRs are also activated by damage-associated molecular patterns (DAMP). As downstream targets of these patterns, the NF-κB and MAPK signalling pathways are activated, ultimately leading to excessive secretion of proinflammatory cytokines and further recruitment of proinflammatory cells, thereby promoting the occurrence and development of PC [117–119]. Additionally, blocking TLR activation inhibits the interaction between NF-κB and MAPK signalling pathways, and directly inhibits activation of TLR4 and TLR7[141]. Pushalkar et al. showed that, in a mouse model of PC, depletion of the intestinal microbiota was associated with immunogenic reprogramming of the pancreatic TME, including a reduction in myeloid-derived suppressor cells, an increase in M1 macrophage differentiation, and an increase in CD4 + T cell Th1 differentiation and CD8 + T cell activation [139]. In addition, the deletion of homologous trimer type 1 collagen in the pancreatic cancer gene engineering mouse model changed the tumours microbiome, increased T cell infiltration, enhanced anti PD-1 treatment, and ultimately inhibited tumour growth [142].

### Interactions between intestinal microbiota and anticancer therapies

With the maturation of microbial therapeutic technologies such as oral probiotics/prebiotics preparations and FMT, numerous studies have shown that the intestinal microbiota and its products can influence the efficacy and toxicity of chemotherapy and immunotherapy in gastrointestinal tumours [8, 143].

### The impact of the intestinal microbiota on chemotherapy efficacy and toxicity

Chemotherapy involves using chemotherapeutic agents to treat cancer. These drugs not only have cytotoxic effects toward cancer cells, but also damage normal cells, leading to toxic side effects such as nausea, vomiting, diarrhoea, hair loss, and immunosuppression. In recent years an increasing number of studies have indicated that the intestinal microbiota can influence the efficacy and toxic side effects of chemotherapy drugs [8, 143]. Research has shown that the intestinal microbiota and its products can enhance the effectiveness of chemotherapy. For example, *Lactobacillus* and its metabolites can enhance the therapeutic effect of 5-fluorouracil on colon cancer and reverse 5-fluorouracil resistance [144, 145]. Butyrate can promote the efficacy of oxaliplatin by regulating CD8 T cell function in the TME [146]. Synergistic effects have been observed between butyrate and irinotecan, in that butyrate reduces the half maximal inhibitory concentration (IC50) of irinotecan [147]. The anti-tumour efficacy of oxaliplatin and cisplatin is reduced in germ-free mice or mice treated with antibiotics [148]. In addition, the intestinal microbiota and its products can significantly alleviate the toxic side effects of chemotherapy. Numerous studies have shown that the combination of various probiotics and prebiotics can improve chemotherapy-induced mucositis, reduce the frequency of diarrhoea and abdominal pain, and lower the incidence of postoperative complications such as pneumonia, infections and anastomotic leakage [149–151]. Consistent results have also been observed in animal experiments, where mice with CRC that received probiotics showed reduced upregulation of pro-inflammatory cytokines and less 5-fluorouracil induced colonic mucositis [152]. The intestinal microbiota can also influence the toxicity of chemotherapeutic drugs. SN-38 is the active metabolite of irinotecan. The hepatic glucuronidation enzyme can convert the active SN-38 into the inactive SN-38-G, thereby reducing the gastrointestinal toxicity of the drug. However, gut bacteria can secrete β-glucuronidase, which converts SN-38-G back into SN-38, leading to diarrhoea [153, 154]. Therefore, the use of β-glucuronidase inhibitors can alleviate irinotecan-induced toxic side effects [155]. In conclusion, the intestinal microbiota plays an important role in improving chemotherapy efficacy and reducing toxic side effects. It is anticipated that future research will further elucidate the mechanisms underlying the interactions between the intestinal microbiota and chemotherapeutic drugs, providing more precise and effective treatment strategies to improve the quality of life and therapeutic outcomes of chemotherapy patients.

### The response of the intestinal microbiota to immunotherapy for gastrointestinal tumours

In addition to chemotherapy, immune checkpoint inhibitors (ICIs) are also an important therapeutic strategy in the treatment of gastrointestinal tumours. ICIs are drugs that restore the immune system's ability to attack tumour cells by inhibiting immune checkpoint function. Numerous studies have shown that the intestinal microbiota can modulate anti-tumour immunity and affect the efficacy of cancer immunotherapy [9]. The intestinal microbiota can improve the efficacy of ICIs by reshaping the TME and modulating immune cell function within the TME, and the combination of the intestinal microbiota and ICIs can reduce the occurrence of immune-related adverse events (irAEs) [9, 143]. Multiple studies support this notion. In a murine model of CpG island methylator phenotype (CIMP) CRC, infection with *Enterotoxic Fragile Bacteroides* promoted the recruitment of IFN-γ-producing CD8 T cells, thereby enhancing the efficacy of anti-programmed cell death ligand 1 (Anti PD-L1) therapy [156]. Takeshi et al. also showed that a combination of 11 bacterial strains can enhance the anti-tumour efficacy of ICIs and promote the proliferation of IFN-γ-producing CD8 T cells [157]. Marie et al. demonstrated that tumour cells in antibiotic-treated and germ-free mice had no response to CTLA blockade, but the efficacy of anti–CTLA-4 therapy was improved after oral administration of *Enterotoxic Fragile Bacteroides*, which activated CD4 T cells and promoted dendritic cell maturation [158]. Gopalakrishnan et al. found that patients who responded to PD-1 treatment had a higher abundance of *Clostridium* in the intestine, as well as exhibiting more T cells within the tumour and higher levels of circulating T cells that kill abnormal cells. Conversely, patients with higher levels of *Bifidobacterium* had higher levels of Treg cells, myeloid-derived suppressor cells, and a weakened cytokine response, leading to inhibition of the anti-tumour immune response [159]. The intestinal microbiota can synthesise and transform a large number of small, molecular metabolites, which can then diffuse from the intestine to the body, influencing local and systemic anti-tumour immune responses and enhancing ICI efficacy. Disruption of gut barrier function by ICI therapy can lead to an increase in the intestinal microbiota and metabolite translocation. Adenosine is a purine metabolite produced by *Bifidobacterium mucosum* and *Bifidobacterium pseudolongum*. Adenosine translocation can activate anti-tumour T cells and enhance ICI efficacy [160]. Synergism between SCFAs and ICIs is currently an area of intense research interest. SCFAs can improve the efficacy of ICI therapy by directly promoting the cytotoxic effects of CD8 T cells, regulating tumour signalling pathways, and reducing the expression of inflammatory factors [9, 161, 162]. In recent years, some studies have indicated that FMT may influence ICI efficacy. Bertrand et al. found that transplantation of faeces from tumour patients who responded to ICI therapy into tumour-bearing mice improved the efficacy of ICI therapy [163]. ICI therapy, which activates the patient's own immune system to attack tumour cells, may also induce the irAEs, for example by inducing immune overactivation and intestinal inflammation. These adverse reactions may limit treatment effectiveness and tolerability. Studies have shown that modulation of the intestinal microbiota can alleviate irAEs associated with ICI therapy, improve treatment tolerance, and enhance treatment efficacy[164]. In patients receiving anti–PD-1 therapy, the abundance of *Bacteroides* was positively correlated with colitis resistance, while *Ruminococcus* abundance was associated with a favourable clinical response and reduced incidence of colitis [165]. Furthermore, a meta-analysis and bioinformatics analysis of four published datasets on ICI-treated tumours conducted by John et al. showed that the *Actinobacteria phylum* and the *Lachnospiraceae/Ruminococcaceae* families of *Firmicutes* were associated with a favourable clinical response and a lower incidence of colitis, while *Streptococceae spp.* were associated with significant irAEs [166]. Sun et al. demonstrated that oral administration of lactobacillus can enhance the expression levels of tight junction protein zona occludens protein 1 (ZO-1) and mucoprotein2 in the colonic mucosa of mice with colitis. This reduces the severity of inflammation and restores intestinal epithelial barrier function[167]. Renga et al. found that indole-3-carboxaldehyde (3-IAld), directly delivered in the intestine, activated the AhR/IL-22 pathway for epithelial barrier function and immune homeostasis during colitis. In addition, 3-IAld also can increase the expression of ZO-1, thereby reducing the severity of inflammation and restoring intestinal epithelial barrier function[168]. In summary, the intestinal microbiota and its products can enhance the efficacy of chemotherapy and immunotherapy for gastrointestinal tumours, while mitigating the adverse reactions caused by treatment. However, current research on the role of gut microbiota and its metabolites in mitigating adverse reactions caused by ICI therapy is primarily focused on exploring correlations, with a lack of in-depth mechanistic studies. Therefore, further mechanistic studies can be considered as a future research direction.

### Modulation of the intestinal microbiota as a therapeutic strategy

In-depth study of the mechanisms by which the intestinal flora regulates gastrointestinal tumours has attracted considerable attention in recent years regarding microbial treatment strategies such as oral probiotics, prebiotics formulations, FMT and antibiotic. These approaches aim to regulate intestinal microbiota balance, enhance immune responses, and promote the production of beneficial metabolic products. These effects could potentially play a positive role in the treatment and prevention of gastrointestinal tumours (Table 4) [169, 170].

**Table 4:** Therapeutic potential and health benefits of probiotics, prebiotics and FMT

Type	Probiotics/Prebiotics/FMT/ Antibiotic	Treatment	Reference
Postoperative patients with colorectal cancer (Placebo group, n = 80 Probiotics group, n = 84)	*Lactobacillus acidophilus,* *Lactobacillus plantarum,* *Bifidobacterium lactis,* *Saccharomyces boulardiisi*	The probiotics group reduced the incidence of postoperative pneumonia, surgical site infection and anastomotic leakage	[171]
Postoperative patients with colorectal cancer (Placebo group, n = 30 Probiotics group, n = 30)	*Bifidobacterium ongum*, *Lactobacillus acidophilus*, *Enterococcus faecalis*	Probiotic therapy reduced the incidence of diarrhea and bacteremia in colorectal cancer patients	[172]
Colorectal model mice (5-fluorouraci group, n = 12 Lcr35group, n = 12 LaBi group, n = 12)	*Lcr35(Lactobacillus casei variety rhamnosus)* *LaBi (Lactobacillus acidophilus, Bifidobacterium bifidum*)	Oral administration of probiotics Lcr35 and LaBi in mice can improve chemotherapy induced intestinal mucositis and inhibit the pro-inflammatory factor TNF in a mouse model- α、 IL-1 β Upregulation of IL-6	[173]
Patients with colorectal cancer, n = 34 (Placebo group, n = 15 Treatment group, n = 19)	*Lactobacillus rhamnosus GG*, *Bifidobacterium lactis Bb12* and inulin enriched with oligofructose	Compared with the placebo group, the content of interferon gamma in the treatment group was higher than that in the placebo group	[174]
Colorectal model mice, n = 20 (Control group, n = 10 GOS group, n = 10)	Galacto-oligosaccharides (GOS)	Compared with the control group, the GOS group had fewer colorectal tumours, increased *Bifidobacteria* and short-chain fatty acids and decreased pro-inflammatory flora	[175]
Colorectal model mice, n = 16 (Control group, n = 8 Lactulos group, n = 8)	*Lactulose*	Compared with the control group, the number of colorectal tumours in *Lactulose* group was less, the degree of inflammation and fibrosis was less, the abundance of *Lactobacillus intestinalis*, *Lactobacillus murinus* and *Bacteroides caecimuris* was significantly increased, and the abundance of *Mucispirillum schaedleri* was significantly decreased	[176]
Colorectal model mice, n = 20 (Control group: FOLFOX (5-fluorouracil, leucovorin, and oxaliplatin), n = 10 Treatment group: FOLFOX + FMT, n = 10)	FMT	FMT alleviated diarrhea and intestinal mucositis caused by FOLFOX treatment, and restored the damaged faecal-intestinal microflora	[177]
Patients with gastric cancer undergoing chemotherapy, n = 24 (Control group: Autologous FMT, n = 12 Treatment group: Allogeneic FMT, healthy donor, n = 12)	FMT	FMT from healthy obese donors improved disease control rate and survival rate in patients with metastatic cancer	[178]
Colorectal model mice, n = 39 (Control group, n = 20 Metronidazole group, n = 19)	Metronidazole	The treatment with metronidazole reduced the abundance of Clostridium and suppressed tumour cell proliferation and tumour growth in a CRC xenograft mouse model	[179]
liver cancer (primary liver cancer cases, n = 1195 matched controls, n = 4640)	Antibiotic	Ever-use of prescription antibiotics was associated with a slightly increased risk of liver cancer, compared to non-use (OR = 1.22, 95% CI = 1.03–1.45)	[180]

Abbreviations: FMT, Faecal Microbiota Transplantation; TNF, Tumour necrosis factor; IL, Interleukin

### Probiotics as a treatment target

Probiotics are a group of active microorganisms that colonise the gastrointestinal tract and are beneficial to the host. They primarily impact the development of gastrointestinal tumours by inhibiting the growth of carcinogenic bacteria, regulating immune function, and protecting mucosal barrier function [169, 181]. *Bacillus*, through the secretion of Bacillus lipopeptides, inhibit colonisation of the gastrointestinal tract by *Staphylococcus aureus* [182]. *Lactobacillus reuteri* ferments glycerol to produce reuterin, which directly inhibits *Clostridium difficile* activity [183]. Raffaella et al. demonstrated that cell-free supernatants from *Lactobacilli* can directly inhibit the activity of pathogenic bacteria. Additionally, the auto-aggregation and co-aggregation properties of *Lactobacilli* promote their colonisation of the gastrointestinal tract while reducing colonisation by pathogenic bacteria [184, 185]. Therefore, probiotics can inhibit the colonization of pathogenic bacteria, reduce the risk of intestinal infections and inflammation, and prevent the occurrence of CRC.

Probiotics have a regulatory effect on the immune system, improving intestinal immune responses, promoting immune cell differentiation, and inhibiting inflammatory responses.. Ma et al. demonstratde that *Lactiplantibacillus plantarum-12*, through the inhibition of NF-κB signalling, alleviated the cancer burden and inflammation in mouse model of AOM/DSS-induced CRC [186]. Similarly, Liu et al. conducted study showing that *Lactobacillus fermentum ZS40* reduced cancer burden and inflammation in AOM/DSS-induced CRC mouse models by suppressing the expression of inflammatory cytokines TNF-α and IL-1β, as well as key proteins IκBα and p65 in the NF-κB signal pathway [187]. *Lactobacillus rhamnosus GG* (LGG) decreased tumour burden in the murine gut cancer models by a CD8 T-cell-dependent manner [188]. Liotti et al. demonstrated that LGG restrains the angiogenic potential of colorectal carcinoma cells by activating formyl peptide receptor 1 [189].

An intact intestinal barrier protects the intestines from damage caused by toxins and pathogens. Studies have shown that strains such as *Lactobacillus rhamnosus GG* and *Lactobacillus plantarum ZJ617* can stimulate mucin production and upregulate the expression of tight junction proteins (claudin-1, occludin, occludens-1, occludens-2) to restore the damaged intestinal mucosal barrier [190–193]. The studies conducted by Oh et al. and Ma et al. revealed that *Lactobacillus gasseri* 505 and *Lactiplantibacillus plantarum*-12 can upregulate the expression of tight junction proteins (claudin-1, occludin, and ZO-1) in tumour tissues of AOM/DSS-induced CRC mouse models [186, 194]. Probiotics have emerged as a viable microbial therapeutic tool. In recent years, oral probiotic preparations have become an important strategy in the treatment of gastrointestinal tumours. Kotzampassi et al. administered *Lactobacillus acidophilus*, *Lactobacillus plantarum*, *Bifidobacterium lactis*, and *Saccharomyces boulardii* capsules to patients with CRC (*n* = 124) and found that, compared with the placebo group, patients in the probiotic group had reduced postoperative pneumonia, surgical site infection, and anastomotic leakage rates, as well as shorter hospital stays [171]. Another study, by Yang et al., showed that the combination of Bifidobacterium longum, *Lactobacillus acidophilus* and *Enterococcus faecalis* reduced the incidence of diarrhoea and bacteraemia in patients with CRC (*n* = 60) [172]. In a mouse model of CRC (*n* = 36), oral administration of the probiotics Lcr35 (*Lactobacillus casei variety rhamnosus*) and LaBi (*Lactobacillus acidophilus* and *Bifidobacterium bifidum*) improved chemotherapy-induced (5-fluorouracil) intestinal mucositis and suppressed upregulation of the pro-inflammatory cytokines TNF-α, IL-1β, and IL-6 [173]. In conclusion, probiotic therapy is an important treatment strategy in the management of gastrointestinal tumours.

### Prebiotics as therapeutic targets

Prebiotics are substances that promote the growth of intestinal probiotics and enhance their activity. They are not degraded by human digestive enzymes and are fermented by the microbiota in the gastrointestinal tract, providing nutritional and energy support for the growth and metabolic activities of probiotics [195]. Prebiotics can promote probiotic colonisation and proliferation, inhibit colonisation by pathogenic bacteria, exert anti-inflammatory effects, and modulate the immune system [196]. Common prebiotics include fructans, galacto-oligosaccharides (GOS), and lactulose [196]. Fructans include fructo-oligosaccharides and inulin. Fructans are beneficial dietary fibres that contribute to increased faecal bulk, alleviate constipation, and promote digestion. Consumption of inulin-type fructans can promote *Bifidobacterium* growth and enhance satiety [197]. The systematic review conducted by Mazraeh et al. suggested that inulin can improve the viscosity of feces of CRC patients after chemotherapy and increase the content of butyrate in their feces [198]. Moreover, fructans can stimulate cytokine release. Monika et al. demonstrated that, compared with the placebo group, CRC patients who consumed a combination of *Lactobacillus rhamnosus GG*, *Bifidobacterium lactis Bb12*, and inulin enriched with oligofructose had increased levels of IFN-γ in their peripheral blood [174]. GOS is an oligosaccharide composed of galactose molecules that can promote *Bifidobacterium* growth [199]. Javier et al. found that, compared with the control group, rats in the GOS group had fewer colorectal tumours, increased levels of *Bifidobacterium* and SCFAs, and decreased abundance of pro-inflammatory microbiota components[175]. Additionally, Qamar et al. demonstrated that the combined application of galactooligosaccharides (GOS) and inulin in AOM/DSS-induced CRC mouse models can effectively reduce the occurrence of aberrant crypt foci and promote the production of SCFAs [200]. Lactulose, a galactose-fructose disaccharide, is derived from lactose by heating or isomerisation. Hiraishi et al. showed that, compared with the control group, mice in the lactulose group had fewer colorectal tumours, lower degrees of inflammation and fibrosis, significantly increased abundance of *Lactobacillus intestinalis*, *Lactobacillus murinus* and *Bacteroides caecimuris*, and significantly decreased abundance of *Mucispirillum schaedleri* [176]. Currently, prebiotics are added to various food products such as biscuits, candies, yogurts, and infant formulas [201]. Increasing the intake of prebiotics in one’s daily diet can support gut health and help prevent and improve certain Symptoms of gastrointestinal tumours.

Faecal microbiota transplantation as a therapeutic strategy.

FMT is a treatment method that involves the transfer of beneficial microbiota from faeces from a healthy individual to the intestines of a patient to restore the balance of the intestinal microbiota and improve treatment efficacy in gastrointestinal cancer patients [202]. According to the 2013 Guidelines for the Treatment of *Clostridium difficile*, FMT has been approved as a clinical method for treating recurrent *Clostridium difficile* infection [203]. In recent years, there has been growing interest in the potential of FMT to treat gastrointestinal cancers and other diseases [202]. FMT can improve intestinal mucosal barrier function and alleviate intestinal mucosal inflammation. Additionally, FMT can restore disruption of the intestinal microecological balance in patients, increasing the abundance of beneficial microbiota components and inhibiting the growth of harmful microbiota components. In a mouse model of CRC (*n* = 20), FMT alleviated diarrhoea and intestinal mucositis caused by FOLFOX (5-fluorouracil, leucovorin, and oxaliplatint) treatment and restored disrupted faecal-intestinal microbiota composition [177] Nicolien et al. treated 24 GC patients undergoing chemotherapy with FMT and found that allogenic FMT (from healthy donors, *n* = 12) improved disease control and survival rates in patients with GC and metastatic cancer compared with autologous FMT (*n* = 12) [178]. FMT has shown promise in treating gastrointestinal tumours, but it is important to note that this therapy also carries potential risks and hazards. Potential risks of FMT include transmission of infectious diseases, adverse reactions, and cross-infections due to improper performance of the procedure. Some case reports have documented infections following FMT, including norovirus gastroenteritis [204], *Escherichia coli* bacteraemia [205] and cytomegalovirus infection [206]. Therefore, when performing FMT, individualised treatment plans should be formulated, and strict adherence to operating protocols is necessary.

### Antibiotic as a therapeutic strategy

Antibiotics are natural compounds produced by microorganisms such as bacteria or fungi. Antibiotics can inhibit the growth and reproduction of bacteria and also interfere with cell growth [207]. Numerous studies have shown that antibiotics have the potential to promote apoptosis, antiproliferation, and anti-metastasis in gastrointestinal tumours, making them a potential treatment option [207]. Bullman et al. demonstrated that treatment with metronidazole reduced the abundance of Clostridium and suppressed tumour cell proliferation and tumour growth in a CRC xenograft mouse model [179]. Similarly, Zackular et al. found that treatment with metronidazole, streptomycin, and vancomycin significantly reduced tumour numbers in an AOM/DSS-induced CRC mouse model [208]. In addition, Zhou et al. showed that salinomycin induced apoptosis in cisplatin-resistant CRC cells through the accumulation of reactive oxygen species [209]. However, an increasing body of evidence suggests that while antibiotics can eliminate pathogenic bacteria, they also disrupt the probiotics, leading to gut dysbiosis and promoting the development of gastrointestinal tumours. Clinical evidence has shown that antibiotic treatment reduces the efficacy of immune checkpoint inhibitors and patient survival rates [210, 211]. A case–control study also indicated an increased risk of cancer among individuals who received antibiotic treatment compared to those who did not (OR = 1.22, 95% CI = 1.03–1.45), although the relationship between antibiotic dosage and cancer risk remains uncertain [180]. Corty et al. demonstrated that antibiotic use in PC patients undergoing chemotherapy increased the risk of anaemia, thrombocytopenia, leukopenia, neutropenia, and gastrointestinal adverse events [212]. In summary, antibiotics are a double-edged sword. While they can be used to treat gastrointestinal tumours, they may also disrupt the gut microbiota and promote the development of such tumours. Therefore, it is important to use antibiotics selectively, aiming to eliminate pathogens while minimizing disruption to the normal gut microbiota, in order to reduce side effects and improve treatment efficacy.

## Future directions and Conclusion

As genomics technology continues to advance, the correlation between the intestinal microbiota and gastrointestinal tumours has become increasingly clear. However, a series of issues still need to be addressed. Numerous studies have identified elements of the intestinal microbiota as potential early screening biomarkers for gastrointestinal tumours. Yet, most of these studies employed different sequencing methods and had small sample sizes (< 100), making it difficult to obtain consistent results. Therefore, it is necessary to establish a multicentre, multi-omics, standardised, and large-scale intestinal microbiota dataset to develop more accurate, reliable, and sensitive early screening methods. Next, the ways in which microbiota influence tumour development through metabolites, immune regulation, and changes in the TME still require in-depth study. Leveraging a combination of genomics, transcriptomics, metabolomics, proteomics, and basic experimental approaches will help uncover the molecular mechanisms underlying the relationship between microbial communities and gastrointestinal tumours. In addition, the development and optimisation of personalised treatment strategies are crucial future research directions. Although the concept of personalised microbiota-related treatments has been proposed, a series of studies is still required to determine the optimal treatment methods, dosage, and time windows. Long-term clinical observation and follow-up are also necessary to assess the effects and safety of personalised treatment. In summary, future research should focus on the complex interactions between the intestinal microbiota and gastrointestinal tumours, as well as exploring methods and norms for personalised microbiota-related therapies. Interdisciplinary collaboration and advanced technological approaches are required to address these issues and challenges.

The intestinal microbiota plays a crucial role in gastrointestinal tumours, and its composition and function are essential for maintaining intestinal health and preventing tumorigenesis. Gastrointestinal tumours include CRC, GC, PC, EC, and more. The intestinal microbiota differs according to tumour type, and dysbiosis of the intestinal microbiota promotes gastrointestinal tumour development through secretion of harmful metabolites, alteration of immune system regulation, and secretion of protein toxins. The intestinal microbiota has also received much attention as a potential therapeutic target for gastrointestinal tumours. the interaction between the intestinal microbiota and anticancer treatments is a key research focus, as the intestinal microbiota and its metabolites can enhance the effectiveness of gastrointestinal tumour chemotherapy and immunotherapy while reducing the incidence and severity of adverse reactions. Additionally, Oral probiotic and prebiotic formulations can suppress tumour growth through restoration of intestinal homeostasis, alleviation of mucosal inflammation, protection of the intestinal barrier, and regulation of the immune system. FMT, an emerging treatment strategy, improves tumour treatment efficacy by transplanting the intestinal microbiota from healthy donors to patients. In conclusion, the intestinal microbiota plays a crucial role in gastrointestinal tumours, and an in-depth understanding of its composition, function, and mechanism will provide important breakthroughs in the development of individualised treatment strategies, as well as the prevention, treatment and early screening of gastrointestinal tumours.

## Data Availability

Not applicable.
